# Synthesis of Acids and Amino Acids by Selective Oxidation of Primary Hydroxyl Groups to Carboxylic acids in Sugars

**DOI:** 10.5539/ijc.v13n2p17

**Published:** 2021-06-30

**Authors:** Natarajan Raju, Rolf E. Swenson

**Affiliations:** 1 National Heart, Blood & Lung Institute/ National Institute of Health, USA

**Keywords:** Selective Oxidation, Sugar amino acids (SAAs), Disaccharides, Trichloroisocyanuric acid (TCICA), TEMPO

## Abstract

Preferential oxidation of primary hydroxyls in unprotected sugars and sugar amino acids is reported using inexpensive and readily available reagents. This method offers a specific oxidation protocol for a variety of carbohydrates. The stereochemical integrity of the starting materials was preserved and a simple workup yielded the products in good yields with high purity. The procedure is compatible with base sensitive groups like Fmoc. Both mono and disaccharides undergo oxidation regioselectively.

## 1. Introduction

Peptide drug candidates have inherent drawbacks such as poor bioavailability, solubility, and oral stability, as well as shorter elimination times (Yusuf et al, 2018). These are typically addressed by the incorporation of unnatural amino acids such as amino acids derived from sugars (SAAs), various linkers, and other scaffolds. When introduced into the peptide chain, SAAs not only enhance the solubility in water but dramatically alter the H-bonding and folding of the peptide chain due to their rigidity and availability of additional hydroxyl groups (Yashoda et al, 2016). In addition, SAAs play a vital role in nature occurring as antibiotics, glycosidase inhibitors, and have been used as structural constraining entities (Martin et al, 2013; Chakraborty et al 2002; Gruner et al 2002; Lohof et al, 1999; Elsa et al, 2001; Jacquelyn &Thomas 2002). Many SAAs have been exploited as templates and synthons in the design of peptidomimetics of biologically active peptides (Frank et al, 2002; Sibylle et al, 2001). NMR studies have shown that cyclo-[Phe-Pro-Phe-D-Trp-Lys-Thr-], adopted a β-turn when pyranoid SAAs replaced the dipeptide, Phe-Pro (Erich *et al*, 1996). Another report revealed that the introduction of a furanose SAA in somatostatin analogs caused apoptosis in both drug-sensitive and multi-drug resistant tumor cells (Sybille et al, 2001). When D-Phe-Val was replaced with an SAA in the sequence, cyclo[-Arg-Gly-Asp-D-Phe-N(Me)Val, targeting integrin receptors, the peptidomimetic showed high affinity for one sub-type of integrins (Renate et al, 2003). Recently published work indicated that the introduction of an SAA in a linear peptide chain as a linker not only improved its solubility but also provided an approach to probe caspase inhibitor activity using molecular imaging techniques without compromising its biological activity (Helen et al, 2013). Sugar amino acid scaffolds have been also utilized in cyclic peptide chains (Johan and Nilsson, 2005) to create an artificial receptor, suggesting that SAAs have enormous potential in artificial receptor designs. These considerations warrant a simple procedure and easy access to sugar amino acids from readily available polyhydroxy amino alcohols/amino sugars.

## 2. Results and Discussion

During our work on the synthesis of peptide libraries, we also became interested in the use of SAAs as linkers or scaffolds to improve the pharmacological profiles and solubility of peptides. SAAs could provide a handle to conjugate radioactive tracers or dyes through the N-terminus or C-terminus to study *in vivo/in vitro* binding abilities of the SAAs containing substrates to their intended targets (Roland et al, 2004). Unfortunately, the SAAs of 2-deoxy-2-amino saccharides are not commercially available and require multistep protection and deprotection strategies to prepare the Fmoc-amino protected acids amenable for automated synthesis (Laiqiang Ying & Jacquelyn Gervay-Hague, 2004). Extensive efforts in this area are detailed (Guillaume et al, 2017), employing TEMPO as the catalyst and various oxidizing agents. However, these reported procedures use a wide range of temperatures, solvents, and laborious workups with inconsistent yields. They are also limited to the oxidation of polysaccharides bearing no base-sensitive groups. Other reported methods for these transformations involve, either the use of expensive oxidizing agents (Kochkar et al, 2000) and/or temperature control or procedures that were again not compatible with base- and acid-sensitive functionalities in the molecule (Arian et al, 1995). These limitations were nicely addressed in a recent report, offering an alternative route to these sugar acids (Mahipal et al, 2018). Surprisingly, this report does not include the preparation of SAAs and in some cases failed to yield the required carbohydrate acids. After scouring the literature for an efficient method to access SAAs, we came across a potential protocol that used trichloroisocyanuric acid (TCICA) in combination with TEMPO and NaBr as catalysts (Lidia et al 2003) to oxidize primary hydroxyls to the corresponding carboxylic acids. However, this procedure also oxidized secondary hydroxyls to the corresponding ketones making it less attractive for oxidation of carbohydrates. This method did not also explore the oxidation of Fmoc-amino alcohols to their respective acids, required for automated peptide synthesis. We believed that the oxidation of N-protected amino sugars with TCICA/TEMPO to produce SAAs could be achieved by careful manipulation of reaction conditions and reactants without the need to protect the hydroxyls. The oxidizer is economical and easier to handle compared to NaOCl/NaClO_2_, which require precise concentrations and are unstable at RT in aqueous solutions over long periods. Initial attempts to prepare the known Fmoc sugar amino acid derived from galactose (Roland et al, 2004) were not successful. The published oxidation method resulted in an inseparable mixture of products though the expected material was one of the major components offering some encouragement. Careful examination of the crude reaction mixture by LC/MS revealed the presence of the expected acid [M+H, 430] and other products with mass units of 432 [M+H], 428[M+H], 464 [M+H], and 508 [M+H] plus the unreacted starting material. Initial oxidation of hydroxymethyl to aldehyde followed by the formation of hemihydrate could result in M+H 432, and further oxidation of the aldehyde to acid followed by another oxidation of a hydroxyl to ketone could explain the mass of 428 [M+H]. We surmised that the mass units 464 and 508 could result from N-halogenation of the Fmoc-amino acid under acidic conditions similar to acetamide by dihaloisocyanuric acid (Scott, 2007). This assumption was confirmed, when the crude mixture was treated with sodium bisulfite for 30 min and reanalyzed. LC/MS showed the disappearance of peaks corresponding to 464 and 508, enhancing the signal intensity of the desired acid [M+H 430]. When the pH of the reaction was carefully monitored, it suddenly dropped to about 2.0 during the addition of TCICA. This observation was also noted during the oxidation of polysaccharides using NaOCl/TEMPO by Arian et al (1995). Lower pH coupled with the reduced solubility of NaHCO_3_ at 0°C in acetone/water might result in increased concentrations of HOCl/HOBr in the reaction, contributing to other side products observed. After exploring various ratios of the oxidizing agent, catalysts, and base, we were gratified to discover that substantially increasing the amount of bicarbonate and controlling the amounts of catalysts and the oxidizer achieved the desired results at RT. We also found that using water/acetonitrile instead of water/acetone as the reaction solvent shortened the reaction times with the diverse sugars evaluated. The use of water-miscible solvents like dioxane, DME, and/or THF seemed to promote N-halogenation. Completion of the reaction was observed in less than 2h in all cases examined. Monitoring the progress of the reaction by LC/MS indicated that the required acid was the major component (>90%) with less than 5% of starting material and other impurities. The other major product was the reduced oxidizing agent, cyanuric acid.

The following table summarizes our initial results with different carbohydrate substrates. 2-Acetamido-2-deoxy-D-glycopyranosides are the most observed structural units in oligosaccharides and glycoconjugates and are associated with a wide range of biological processes (Raymond 1996). Hence, we selected some common 2-acetamidopyranoses (Entries 1–3, Petrovic et al, 2006) for our oxidation procedure and were delighted to find that they underwent clean oxidation to provide the acids as their sodium salts with high purities. Next, we explored the oxidation of C_1_-nitrophenyl pyranoses (Entries 3–6). These derivatives have been extensively used to study enzyme activities of β-glycosidases (Elaine et al, 2016) that are involved in the natural degradation of sugars. In addition, the reduction of the nitro group followed by Fmoc protection of the amine would offer a range of unnatural SAAs, that could be exploited as linkers or scaffolds in peptide libraries. Oxidation was again very clean and yielded the corresponding acids as their sodium salts in good isolated yields and with no epimerization at the C_1_ (Rajendra et al, 1978). Entry 7 was reported as an intermediate for the synthesis of a radiolabeled ligand to elucidate the activities of the enzyme *Caspase-3* (Helen et al, 2013). Our procedure produced the desired carboxylic acid in good isolated yields with a simple column chromatography compared to the reported procedure (Roland et al, 2004). Finally, disaccharides were subjected to this oxidation protocol and found to undergo oxidation to carboxylic acids without any notable side products (Entries 8 & 9; Madeleine et al, 2011 and Kobayashi et al, 1994), which may prove useful in the design of new heparin mimics (Danzhou et al, 1994). The products from the oxidation were supported by observed analytical data. Both the proton and ^13^C NMRs conclusively proved that the configuration was retained at the susceptible C_1_ carbon of the sugars.

## 4. Conclusion

This optimized method offers a convenient approach to sugar-derived carboxylic acids and amino acids from commercially available unprotected substrates. Base sensitive groups like Fmoc- and C_1_-4-nitrophenoxy were well tolerated in our procedure. This oxidation protocol does not require temperature control, uses an economical oxidizing agent and standard column chromatography is sufficient to isolate the products in good yields. Recent work (Thomas et al, 2019) demonstrates that peptide chains could be assembled using 1-chloro-3,5-dimethoxy triazine as the coupling agent, which tolerates the unprotected hydroxyls. Current work reported here could provide an efficient tool to prepare SAAs suitable for automated synthesis. Work is in progress to synthesize other sugar-based amino acids for incorporation into peptide libraries.

## 5. Experimental

### General:

NMR data were recorded on a Varian 400 MHz NMR spectrometer. Mass spectra were obtained on an Agilent 1200 series instrument under ESI conditions. High-Resolution Mass Spectral data were recorded on a Varian Xevo G2-XSQTOF Mass Spectrometer. Purification of the products was carried out with a Teledyne Isco Combiflash® Rf+ instrument. The starting sugars were purchased from Carbosynth, LLC and used as received except for derivative **13** (Roland Haubner, et al 2004). All other chemicals and solvents were procured from Millipore-Sigma and were used as received. The purity of the fractions was checked with Agilent 1200 series LC connected to a Mass Spectrometer. Samples were analyzed by LC/MS under the following conditions; column: Agilent Zobrax RP C_18_; 3.5 microns; 4.6 × 50 mm; elution rate: 1.0 mL/min; gradient: 0% B for 3min and then ramped up to 100%B over 5 min; solvent A-water with 0.1% TFA (v/v) and solvent B- acetonitrile with 0.1% TFA (v/v); detection @ 210nm

#### General procedure for oxidation (representative example; detection @210 nm)

Methyl 2-acetamido-2-deoxy-α-D-mannopyranose (0.168g, 0.5 mmol) was dissolved in 5.0 mL of water and 1.0 mL of acetonitrile. Powdered sodium bicarbonate (0.63g, 7.5 mmol) was added and the mixture was stirred vigorously. Sodium bromide (0.025 mmol, 2.5 mg) and TEMPO (0.8mg, 0.005 mmol) were added and stirring was continued. Trichloroisocyanuric acid (0.244g, 1.05 mmol) was added in portions over 5 min. with vigorous stirring at RT. The reaction was followed by LC/MS and after completion, 0.5 mL of ethanol was added to the reaction mixture, and stirring was continued for 1h. The crude mixture was diluted with 50.0 mL of water and freeze-dried. The resulting solid was dissolved in a minimal amount of water, filtered through a 0.2m filter and the product was isolated via reverse phase (C_18_) flash chromatography. Yield: 108.0 mg (80%)

#### Purification of sugar acids

Method A (Products 2,4,6,8,12,16 & 18): The crude product in about 5.0 ml of water was injected onto a 275.0g flash C_18_ RP column (RediSep® R_f_ High-Performance Gold) and eluted with water until all the expected products were eluted. Elution rate: 60.0 ml/min; Detection @ 210 nm. Pure fractions were collected and freeze-dried to yield the acids as fluffy colorless solids.

Method B (Product 14): A linear gradient of 0–100% acetonitrile in water over 30 min was used with detection @ 280nm. Pure fractions (LC/MS) were collected and freeze-dried to yield the product as a colorless solid.

#### Analytical Data for the products

Sodium (2S,3S,4R,5R,6R)-5-acetamido-3,4-dihydroxy6-methoxytetrahydro-2H-pyran2-carboxylate (**2**): (Method A; 68%); m. p. 158–160°C (m/d); ^1^H NMR (Deuterium Oxide, 400 MHz): δ (ppm) 4.52 (d, *J*=8.5 Hz, 1H), 4.02 (d, *J*=9.6 Hz, 1H), 3.81 – 3.71 (m, 1H), 3.69 – 3.55 (m, 2H), 3.51 (s, 3H), 2.05 (s, 3H); ^13^C NMR (101 MHz): δ (ppm) 174.69, 172.16, 101.95, 74.38, 73.35, 71.46, 57.26, 55.04, 22.06. M. S. [M+H] 250.1; HRMS: Calcd for C_9_H_14_NO_7_ (M-H) 248.0770; Found: 248.0770

Sodium (2S,3R,4R,5R,6S)-5-acetamido-3,4-dihydroxy-6-methoxytetrahydro-2H-pyran-2-carboxylate (**4**): (Method A; 75%); m. p. 183–187°C (m/d); ^1^H NMR (Deuterium Oxide, 400 MHz): δ (ppm) 4.84 (d, *J*=3.7 Hz, 2H), 4.19 (dd, *J*=11.1, 3.7 Hz, 1H), 3.95 (dd, *J*=11.1, 3.3 Hz, 1H), 3.39 (s, 3H), 2.06 (s, 3H); ^13^C NMR (101 MHz): δ (ppm) 175.63, 174.61, 98.06, 71.27, 69.90, 67.93, 55.28, 49.55, 21.90.; M. S. [M+H] 250.1; HRMS : Calcd for C_9_H_14_NO_7_ (M-H) 248.0770; Found: 248.0772

Sodium (2S,3S,4S,5R,6R)-3,4,5-trihydroxy-6-(4-nitrophenoxy)tetrahydro-2H-pyran-2-carboxylate (**6**): (Method A; 80%); m. p. 225–228°C (m/d); ^1^H NMR (Deuterium Oxide, 400 MHz): δ (ppm) 4.25 (dd, *J*=4.5, 1.7 Hz, 1H), 3.88 (dd, *J*=9.7, 4.6 Hz, 1H), 3.81 (d, *J*=9.7 Hz, 1H), 3.60 (t, *J*=9.7 Hz, 1H), 3.31 (s, 3H), 1.96 (s, 3H); ^13^C NMR (D_2_O, 101 MHz): δ (ppm) 176.50, 174.65, 99.90, 73.32, 69.13, 68.86, 54.93, 54.92, 52.04, 21.89. M. S. [M+H] 250.1; HRMS: Calcd for C_9_H_14_NO_7_ (M-H) 248.0770; Found: 248.0777

Sodium (2S,3S,4S,5R,6R)-3,4,5-trihydroxy-6-(4-nitrophenoxy)tetrahydro-2H-pyran-2-carboxylate (**8**): (Method A: 71%); m. p. 235–237°C (m/d); ^1^H NMR (Deuterium Oxide, 400 MHz): δ (ppm) 8.33 – 8.24 (m, 2H), 7.37 – 7.29 (m, 2H), 5.85 (d, *J*=3.d7 Hz, 1H), 4.04 – 3.94 (m, 2H), 3.85 (ddd, *J*=9.8, 3.6, 0.7 Hz, 1H), 3.62 (ddd, *J*=9.8, 8.9, 0.7 Hz, 1H); ^13^C NMR (D_2_O, 101 MHz): δ (ppm) 175.93, 161.36, 142.37, 126.02, 116.70, 96.55, 73.07, 72.64, 71.72, 70.72.; M. S. [M-H] 314.1; HRMS: Calcd for C_12_H_13_NO_9_ (M-H) 314.0512; Found: 314.0512

Sodium (2S,3R,4S,5R,6R)-3,4,5-trihydroxy-6-(4-nitrophenoxy)tetrahydro-2H-pyran-2-carboxylate (**10**): (Method A; 67%); m. p. 258–261° C (m/d); ^1^H NMR (Deuterium Oxide, 400 MHz): δ (ppm) 8.18 – 8.09 (m, 2H), 7.23 – 7.15 (m, 2H), 5.75 (d, *J*=3.7 Hz, 1H), 4.26 – 4.17 (m, 2H), 4.06 (dd, *J*=10.3, 3.4 Hz, 1H), 3.95 (dd, *J*=10.4, 3.7 Hz, 1H); ^13^C NMR (101 MHz): δ (ppm) 174.96, 161.67, 142.18, 126.00, 116.61, 96.79, 72.57, 70.53, 69.41, 67.51; M. S. [M-H] 314.1; HRMS: Calcd for C_12_H_13_NO_9_ (M-H) 314.0512; Found: 314.0516

Sodium (2S,3S,4S,5S,6R)-3,4,5-trihydroxy-6-(4-nitrophenoxy)tetrahydro-2H-pyran-2-carboxylate (**12**): (Method A; 65%); m. p. 275–279°C; ^1^H NMR (Deuterium Oxide, 400 MHz): δ (ppm) 8.36 – 8.29 (m, 2H), 7.42 – 7.35 (m, 2H), 5.90 (d, *J*=2.4 Hz, 1H), 4.29 – 4.23 (m, 1H), 4.18 (dp, *J*=6.3, 2.9 Hz, 1H), 4.06 – 4.00 (m, 2H); ^13^C NMR (101 MHz): δ (ppm) 176.02, 160.99, 151.13, 142.25, 126.00, 116.57, 97.48, 74.07, 70.09, 69.26, 68.67; M. S. [M-H] 314.1; HRMS: Calcd for C_12_H_13_NO_9_ (M-H) 314.0512; Found: 314.0512

Sodium(2S,3R,4R,5R,6R)-6-(((((9H-fluoren-9-yl)methoxy)carbonyl)amino)methyl)-3,4,5-trihydroxytetrahydro-2H-pyr an-2-carboxylate (**14**): (Method B; 73%); m. p. 195–200°C (m/d); ^1^H NMR (400 MHz, DMSO-*d*_6_) δ 7.90 (d, *J* = 7.5 Hz, 3H), 7.71 (d, *J* = 7.3 Hz, 3H), 7.43 (t, *J* = 7.4 Hz, 4H), 7.35 (t, *J* = 7.4 Hz, 3H), 4.31 (d, *J* = 7.3 Hz, 2H), 4.25 (q, *J* = 7.2 Hz, 2H), 4.00 (s, 1H), 3.77 (d, *J* = 16.1 Hz, 2H), 3.58 (dd, *J* = 13.0, 6.4 Hz, 2H), 3.37 (d, *J* = 15.6 Hz, 3H), 3.22 – 3.17 (m, 1H), 3.14 – 3.06 (m, 2H); ^13^C NMR (101 MHz, DMSO-d_6_) δ 172.06, 156.63, 143.90, 140.69, 127.60, 127.12, 125.21, 120.09, 79.45, 78.91, 74.59, 70.68, 68.43, 65.61, 46.68, 42.04; M. S. [M+H] 430.1, [M+Na] 452.2; HRMS: Calcd for C_22_H_22_NO_8_(M-H) 428.1345; Found: 428.1346; Lit: ^13^C NMR (125 MHz, DMSO-d_6_) 170.2 (COOH), 156.2 (Fmoc-CO), 143.9 – 120.1 (aromatic carbons), 78.62, 77.1, 73.8, 70.2, 67.9, 65.4, 46.8 and 42.2

Sodium(2S,3S,4R,5R,6R)-6-(benzyloxy)-3-(((2R,3R,4S,5R,6S)-6-carboxylato-3,4,5-trihydroxytetrahydro-2H-pyran-2-yl)oxy)-4,5-dihydroxytetrahydro-2H-pyran-2-carboxylate (**16**): (Method A, 68%); m. p. 240–245°C (m/d); ^1^H NMR (Deuterium Oxide, 400 MHz): δ (ppm) 7.56 – 7.41 (m, 5H), 4.99 (d, *J*=11.6 Hz, 1H), 4.76 (d, *J*=5.4 Hz, 1H), 4.61 (d, *J*=8.0 Hz, 1H), 4.47 (d, *J*=7.7 Hz, 1H), 4.24 (d, *J*=3.5 Hz, 1H), 4.12 (s, 1H), 3.89 (d, *J*=9.7 Hz, 1H), 3.81 – 3.72 (m, 2H), 3.67 (d, *J*=9.1 Hz, 1H), 3.59 (dd, *J*=9.9, 7.8 Hz, 1H), 3.45 (t, *J*=8.6 Hz, 1H); ^13^C NMR (101 MHz): δ (ppm) 175.16, 174.50, 136.45, 131.17, 128.74, 128.24, 102.73, 100.80, 81.19, 75.67, 75.46, 74.60, 72.55, 71.35, 70.59, 70.04; M. S. [M-H] 459.2; HRMS: Calcd for C_19_H_23_O_13_ (M-H) 459.1139; Found: 459.1143

Sodium(2S,3S,5R,6R)-3-(((2S,3R,4S,5R,6S)-6-carboxylato-3,4,5-trihydroxytetrahydro-2H-pyran-2-yl)oxy)-4,5-dihydro xy-6-(4-nitrosophenoxy)tetrahydro-2H-pyran-2-carboxylate (**18**): (Method A; 73%); m. p. 208–212°C (m/d); ^1^H NMR (Deuterium Oxide, 400 MHz): δ (ppm) 8.32 (d, *J*=9.3 Hz, 2H), 7.36 (d, *J*=9.3 Hz, 2H), 5.88 (d, *J*=3.7 Hz, 1H), 5.67 (d, *J*=3.9 Hz, 1H), 4.26 (t, *J*=9.3 Hz, 1H), 4.01 – 3.91 (m, 2H), 3.77 (t, *J*=9.5 Hz, 1H), 3.65 – 3.57 (m, 1H), 3.47 (t, *J*=9.6 Hz, 1H); ^13^C NMR (101 MHz): δ (ppm) 176.86, 175.08, 161.29, 142.45, 126.08, 116.79, 97.45, 96.43, 75.51, 73.48, 72.98, 72.32, 72.06, 71.43, 70.86; M. S. [M-H] 490.2; HRMS: Calcd for C_18_H_20_NO_15_ (M-H) 490.0833; Found: 490.08839

## Figures and Tables

**Scheme:**
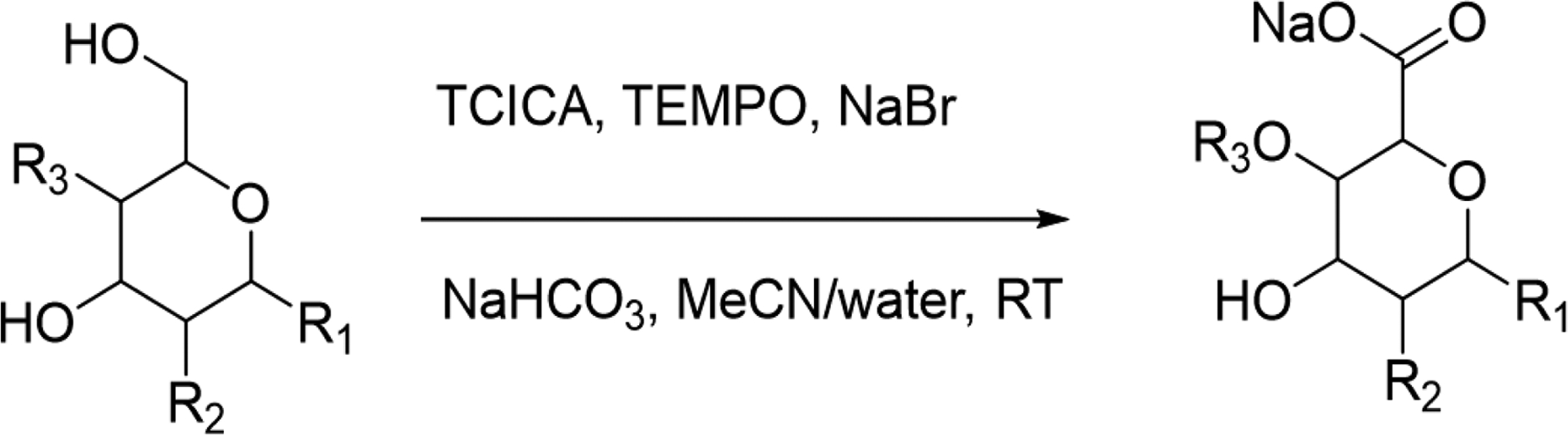


**Table I.** Substrates used for oxidation

Entry	Substrate	Product	Yields^a^/Time in min^b^
1	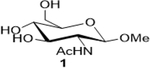	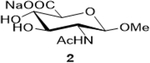	68 (30)
2	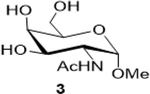	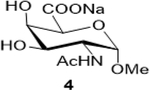	75 (30)
3	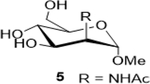	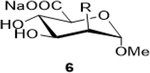	80 (30)
4	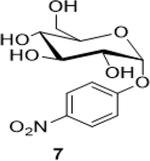	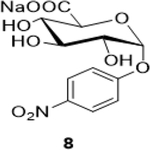	71 (60)
5	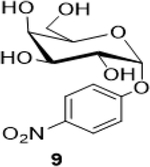	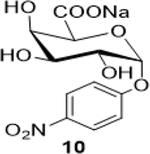	67 (60)
6	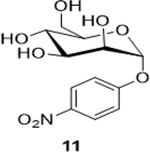	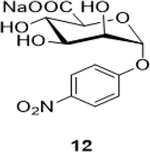	65 (60)
7	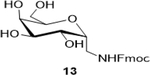	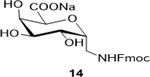	73 (120)
8	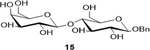	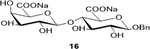	68 (90)
9	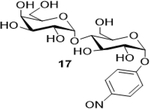	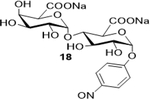	73 (90)

a isolated yield;

b Reaction time
